# HSF1 modulates lipid metabolism and ferroptosis in sarcopenia: a novel diagnostic biomarker and therapeutic target

**DOI:** 10.3389/fmolb.2026.1727633

**Published:** 2026-04-30

**Authors:** Dalu Cheng, Zhitao Shangguan, Xiaoqing Ye, Gang Chen, Qiong Lin, Jiandong Li

**Affiliations:** 1 Department of Clinical Medicine, Fujian Medical University Union Hospital, Fuzhou, Fujian, China; 2 Department of Orthopedics, Fujian Medical University Union Hospital, Fuzhou, Fujian, China; 3 Department of Respiratory and Critical Care Medicine, Fujian Medical University Union Hospital, Fuzhou, Fujian, China

**Keywords:** diagnostic markers, ferroptosis-relevant genes, lipid metabolism, machine learning algorithms, sarcopenia

## Abstract

**Background:**

Sarcopenia, an age-correlated decline in skeletal muscle mass and function, has been increasingly linked to ferroptosis, which is an iron-dependent form of regulated cell death driven by lipid peroxidation. Nevertheless, the diagnostic potential and mechanistic role of ferroptosis-relevant genes (FRGs) in sarcopenia remains poorly understood.

**Methods:**

We integrated bioinformatics analyses and experimental validation to identify and characterize key FRGs in sarcopenia. By employing RNA-seq data from public datasets (GSE25941 and GSE9103), we identified differentially expressed FRGs and applied weighted gene co-expression network analysis (WGCNA) and multiple machine learning algorithms (comprising LASSO, SVM, KNN, XGBoost, and NNET) to screen for diagnostic biomarkers.

**Results:**

Six hub FRGs (*ACSL6*, *CDKN1A*, *ATG4D*, *DECR1*, *HSF1*, and *MIB1*) were identified with AUC>0.8 as robust diagnostic biomarkers, among which HSF1 exhibited the highest diagnostic value (AUC = 0.9247). Molecular docking suggested quercetin and cycloheximide as potential therapeutic agents targeting HSF1. As demonstrated by functional experiments in C2C12 myoblasts, HSF1 silencing facilitated lipid metabolism, elevated ferroptosis markers, and induced muscle atrophy, while its overexpression attenuated these effects.

**Conclusion:**

Our findings not only underscore the significance of FRGs in sarcopenia pathogenesis but also highlight their potential as diagnostic markers and therapeutic targets.

## Introduction

Sarcopenia, accompanied by declining physical function, is a progressive condition characterized by the gradual loss of skeletal muscle mass and strength (Cruz-Jentoft and Sayer, 2019). This age-correlated disease not only markedly impacts mobility but also increases the risk of falls and fractures. It is, furthermore, associated with increased morbidity and mortality and reduced quality of life (Sayer et al., 2024). Its unfavorable prognosis often gives rise to disability, loss of independence, and more substantial healthcare burdens (Papadopoulou, 2020). Combined with nutritional interventions—particularly high-protein diets or supplements like leucine and vitamin D—current treatments center around resistance exercise to stimulate muscle hypertrophy and protein synthesis (Shen et al., 2023; Noor et al., 2021). Pharmacological approaches, such as testosterone or myostatin inhibitors, are under investigation but remain limited (Rolland et al., 2023). These strategies are intended to reinforce muscle regeneration, lessen inflammation, and counteract anabolic resistance (Liu et al., 2025). Driven by factors such as oxidative stress and hormonal changes, the underlying mechanisms involve chronic inflammation (Hashimoto et al., 2023), ferroptosis (Ru et al., 2025; Ding et al., 2021), lipid metabolism (Lee et al., 2020), and impaired satellite cell activity (Wang et al., 2024). Nonetheless, the heterogeneity of sarcopenia necessitates deeper molecular insights. Identifying reliable biomarkers could enable early diagnosis, individualized therapy, and more desirable monitoring of disease progression (Liu et al., 2024a; Liu et al., 2024b; Hsu et al., 2024). Consequently, it is important to explore more molecular targets and develop precision medicine approaches for sarcopenia management.

As an iron-dependent new type of cell death, ferroptosis is triggered by intracellular iron (Jiang et al., 2021). The process involves a diverse spectrum of physiological activities that comprise oxidative stress and the lipid and iron metabolisms (Ma et al., 2022; Zhang HC. et al., 2022). The abnormalities of ferroptosis have been proven to be associated with the development of multiple diseases encompassing cancer (Ma et al., 2022), neurodegenerative disorders (Ryan et al., 2023), cardiovascular diseases (Miao et al., 2023), and sarcopenia (Chen et al., 2024), where it serves as a potential diagnostic and therapeutic target. The diagnostic ferroptosis-associated gene (FRG) signature was identified in osteoporosis (Hu et al., 2022). In acute myocardial infarction, FRGs were screened as diagnostic biomarkers and participated in oxidative stress and inflammatory response (Miao et al., 2023). Chen et al. (2024) identified FRGs as diagnostic and therapeutic targets in sarcopenia. Nonetheless, its role in sarcopenia remains ambiguous, which underlines the necessity for further exploration of ferroptosis-relevant molecular markers in age-correlated muscle wasting.

As suggested by emerging evidence, ferroptosis plays important roles in disease by disrupting lipid metabolism and triggering the apoptosis process. As relevant studies have demonstrated, cardiomyocyte ferroptosis was attenuated by the suppression of glutathione after ischemia–reperfusion, which contributed to lowering myocardial infarct size (Ichihara et al., 2023). The initiation and execution of ferroptosis are regulated by the iron and lipid metabolisms (Wu et al., 2021). The accumulation of lipid peroxides is due to impaired glutathione peroxidase 4 (GPX4) activity and iron overload, which exacerbate cell damage and functional decline (Lai et al., 2022). As evidenced by associated studies, GPX4-mediated ferroptosis could defend against ultraviolet irradiation-induced skin injury (Feng et al., 2022). Despite these insights, the molecular mechanism of specific ferroptosis-relevant biomarkers in sarcopenia remains poorly defined. Further screening and validation of such markers could reinforce early diagnosis and targeted interventions.

Therefore, we constructed an FRG signature and confirmed its diagnostic value for sarcopenia patients. The above goals were realized through comprehensive bioinformatics analysis encompassing WGCNA and machine learning algorithms. Cell experiments were then conducted to further verify the effects of the novel gene on muscle degeneration and lipid metabolism. More importantly, investigating its molecular pathways may uncover novel therapeutic strategies to mitigate sarcopenia progression.

## Methods

### Data source

**FIGURE 1:**
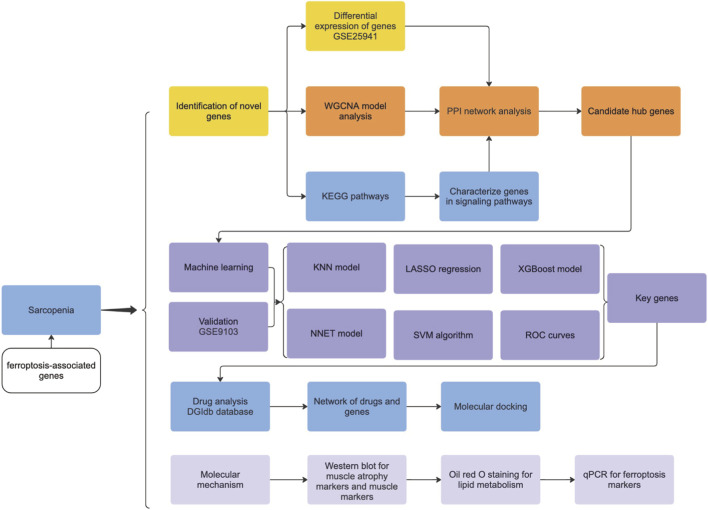
Study’s flow chart.

RNA sequencing data in FPKM format were collected from GSE25941—comprising 15 control samples and 21 sarcopenia samples—as the training database. GSE9103 was used as the testing database. FerrDb is a database dedicated to ferroptosis regulators and ferroptosis–disease associations. The list of FRGs was collected from FerrDb (http://www.zhounan.org/ferrdb/index.html) and previous literature (Zh et al., 2024). Ultimately, 486 ferroptosis-relevant genes (FRGs) were obtained by removing duplicate gene names (Supplementary Table 1). The entire experimental analysis workflow is illustrated in Figure 1.

### Differential expression of FRGs

By utilizing RNA sequencing data of GSE25941 between 15 control and 21 sarcopenia samples, the differential expression of genes was performed by the “limma” R package with P < 0.05 and |log_2_ (fold change, FC)|>1.5. The results were visually presented by referring to heatmaps. A Venn diagram was utilized to select the intersection of the differential expression of genes and FRGs. Subsequently, we performed pathway enrichment analysis on the differential FRGs by employing the “clusterProfiler” R package. The Sankey network between signaling pathways and differentially expressed FRGs was illustrated using Sankey online tools (https://www.sankeymatic.com/).

### Screening key module by WGCNA analysis and protein–protein interaction network

In order to describe patterns of gene co-expression between different samples, the WGCNA R package was utilized to analyze differentially expressed FRGs with clustering subgroups. Prior to network construction, a sample dendrogram and trait heatmap were generated to identify potential outliers. As shown in Figure 3A, one sample (GSM637533) exhibited an exceptionally large average Euclidean distance (>3 standard deviations from the mean) from all other samples within the dendrogram. This significant deviation suggests a substantial global expression profile difference, potentially due to technical artifacts (such as poor RNA quality or batch effects) or an extreme biological state not representative of the study cohort. To ensure the robustness and biological relevance of the co-expression networks derived, this single outlier sample was excluded from the subsequent WGCNA. All analyses for Figure 3 and related downstream network construction were therefore performed on the remaining 35 samples.

Network topology analysis was employed to calculate adjacency between genes with the standard of *R* ^*2*^ = 0.7 to screen the optimal soft threshold (soft threshold = 12) (Liu et al., 2023). Subsequently, a clustering tree diagram was drawn by the hierarchical clustering method to construct the weighted gene co-expression network. The correlation of gene significance (GS) and module membership (MM) of genes were calculated with grouping factor as the clinical phenotype.

In addition, protein–protein interaction (PPI) was established by the STRING database. Cytoscape (version: 3.8.2) software was then employed to visualize the intersection genes between module genes and FRGs.

### Machine learning algorithm

Multiple machine learning methods were employed to screen novel FRGs, encompassing least absolute shrinkage and selection operator (LASSO) regression analysis, support vector machine (SVM), k-nearest neighbor (KNN), extreme gradient boosting (XGBoost), and neural network (NNET). To filter out the significant variables and construct the best classification model, the L1-penalty (lambda) was applied to analyze LASSO regression by employing the “glmnet” R package. The SVM was performed via the R package “e1071.” On the basis of the best K parameter, the KNN algorithm was calculated by the “kknn” R package. XGBoost was utilized to minimize prediction errors by iteratively adding weak learners through building upon decision trees by employing the “xgboost” R package. The NNET was built via the “nnet” R package. A receiver operating curve (ROC) was utilized to assess how the model affects the training data (GSE25941) and validation set (GSE9103). The area under the curve (AUC) was calculated to measure the prediction accuracy of the machine learning model by utilizing the “pROC” R package.

ROC analysis of hub genes was also conducted using plotROC and was visualized by the “ggplot2” package.

### Prediction of candidate drugs for hub genes and molecular docking

The Drug–Gene Interaction database (DGIdb) was employed to explore the potential drugs or compounds that interact with hub FRGs. The drug–gene interaction network was visualized by Sankey online tools (https://www.sankeymatic.com/). Subsequent to the above steps, molecular docking was conducted by employing MGLtools (version: 1.5.7). The HSF1 protein structure (PDB ID: 5HDG) was downloaded from the PDB database (https://www.rcsb.org/). After moving water molecules, the crystal structure was minimized by utilizing an energy minimization automatic program. The small molecule structure (Compound CID of cycloheximide: 6197; Compound CID of quercetin: 5280343) was downloaded from the PubChem database (https://pubchem.ncbi.nlm.nih.gov/). Two small molecules were assigned a CHARMM force field to accurately model its interactions with the HSF1. The parameters were set as per Wang et al. (2025). Vina software was employed to visualize the results.

### Cell culture and transfection

C2C12 cells were purchased from Procell (China) and cultured in Dulbecco’s Modified Eagle Medium (DMEM) with 10% FBS and 1% penicillin/streptomycin under conditions of 5% CO_2_ and 37 °C. HSF1-specific small interfering RNA (siRNA) was sourced from General Biol (China). The siRNA was transfected at a concentration of 50 nM in the presence of transfection reagents by employing the Lipofectamine 2000 (#11668019, Invitrogen, Thermo Fisher Scientific) and Opti-MEM™ medium (#31985070, Invitrogen, Thermo Fisher Scientific), when myoblasts reached 40% confluence. HSF1-overexpression plasmids were purchased from General Biol (China). A total of 10 µg plasmid was used for transfection in the presence of a transfection reagent comprising HSF1-overexpression (oe-HSF1) plasmids and vector (oe-NC) plasmids, when myoblasts reached 70% confluence.

### Western blot analysis

RIPA lysis buffer and phenylmethanesulfonyl fluoride were employed to obtain the total protein of C2C12 cells on ice for 30 min. Subsequent to ultrasonic crushing, the lysate was centrifuged at 16,000 rpm for 30 min. A BCA protein assay kit (#PA115, TIANGEN, Beijing, China) was employed to measure the protein quantification in supernatant. The protein was separated by 10% SDS-PAGE electrophoresis at 80 V for the stacking gel and 120 V for the separation gel. A PVDF membrane (Millipore, IPVH00010) was activated with methanol for 2 min before use. The proteins were transferred onto the PVDF membrane under a constant flow of 120 mA. The membranes were immunoblotted with the diluted primary antibodies overnight at 4 °C subsequent to washing thrice with PBST. The diluted secondary antibody was incubated at room temperature for 60 min. After the PBST washing, ECL Western blot reagent was employed to detect immunoreactivity. The “ImageJ” software was utilized to quantify signal bands through densitometry analysis. The antibodies used were HSF1 (AF0098, Affinity Biosciences), TRAP1 (DF7073, Affinity Biosciences), FBXO32 (DF7075, Affinity Biosciences), Myogenin (DF8273, Affinity Biosciences), ATGL (DF7756, Affinity Biosciences), HSL (AF6403, Affinity Biosciences), SREBP1c (AF6283, Affinity Biosciences), ELOVL6 (DF4039, Affinity Biosciences), and GAPDH (AF7021, Affinity Biosciences).

### RNA extraction and real-time quantitative PCR

In line with the manufacturer’s instructions, total RNA was extracted from cultured cells utilizing TRIzol® reagent (Invitrogen, United States). RNA concentration and purity were determined by utilizing a NanoDrop spectrophotometer (Thermo Fisher Scientific, United States). Complementary DNA (cDNA) was synthesized from 1 μg of total RNA by referring to a PrimeScript™ RT Reagent Kit (Takara, Japan). Quantitative real-time PCR (RT-PCR) was performed with SYBR® Green Premix (Roche, Switzerland) on a QuantStudio 5 Real-Time PCR System (Applied Biosystems, USA). The reaction conditions were as follows: initial denaturation at 95 °C for 30 s, followed by 40 cycles of 95 °C for 5 s and 60 °C for 30 s. Gene expression levels were normalized to GAPDH as an internal control, and relative quantification was calculated by referring to the 2^–ΔΔCt^ method. Quantitative analysis of ferroptosis-relevant genes was performed based on data obtained from RT-PCR experiments. The relative expressions were normalized to the control group and are presented as mean ± standard deviation. All reactions were performed in triplicate to ensure reproducibility. The primer list is presented in Supplementary Table 2.

### Quantification of triglyceride content

Intracellular triglyceride levels were measured utilizing a commercial Triglyceride Quantification Kit (Sigma-Aldrich, USA, Catalog #MAK266) according to the manufacturer’s protocol. In brief, cells were washed with PBS and lysed with an appropriate volume of assay buffer. The lysates were centrifuged at 12,000 × g for 10 min at 4 °C to remove insoluble debris. The supernatant was collected and incubated with a triglyceride reaction mix at 37 °C for 30 min. Absorbance was measured at 570 nm by adopting a microplate reader (BioTek, USA). Triglyceride concentrations were normalized to the total protein content determined by a BCA protein assay kit (Beyotime, China) and expressed as μg per mg of protein. All samples were analyzed in triplicate.

### Oil Red O staining

The details were outlined in Bini et al. (2024). All cells were cultured in 10% formalin for 10 min. After rinsing with distilled water, all cells were immersed in 60% isopropanol for 1 min to reinforce lipid retention. Samples were then stained with filtered Oil Red O (ORO) working solution for 10 min. After rinsing with distilled water, nuclei samples were counterstained with Mayer’s hematoxylin. Clear microphotographs can be taken up in oil immersion.

### Statistical analysis

All data are represented as mean ± SD. All statistical analyses were performed by adopting R software. Student's *t*-test analysis was utilized to calculate differences between two groups, and one-way analysis of variance was used to make multiple group comparisons. P < 0.05 was considered statistically significant.

## Results

### Differential expression of ferroptosis-relevant genes

**FIGURE 2:**
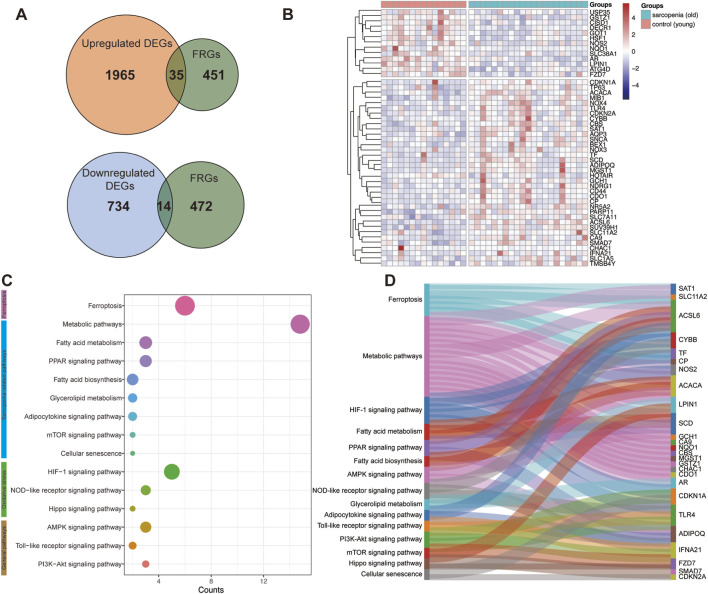
Differential expression of ferroptosis-relevant genes (FRGs) and KEGG enrichment analysis. **(A)** Venn diagram exhibiting intersection with differentially expressed genes (DEGs) and FRGs. **(B)** Heatmap depicting the expression profiles of DEGs. **(C)** KEGG pathways. **(D)** Sankey plot illustrating the network of signaling pathways and genes.

To screen differentially expressed genes (DEGs) between control and old sarcopenia, GSE25941 data were utilized to identify DEGs encompassing 15 control samples and 21 old sarcopenia samples. We obtained 2748 DEGs comprising 2000 upregulated and 748 downregulated genes. A Venn diagram identified 35 overlapping DEGs between upregulated genes and ferroptosis-relevant genes (FRGs) and 14 intersections of downregulated genes and FRGs (Figure 2A). These overlapping DEGs were visually represented through heatmaps (Figure 2B). Functional enrichment KEGG showed these genes to be enriched in multiple signaling pathways, comprising sarcopenia-related, ferroptosis-relevant, and oxidative stress-related pathways (Figure 2C). A Sankey plot illustrates the network of signaling pathways and genes (Figure 2D).

### Identification of hub modules and genes

**FIGURE 3:**
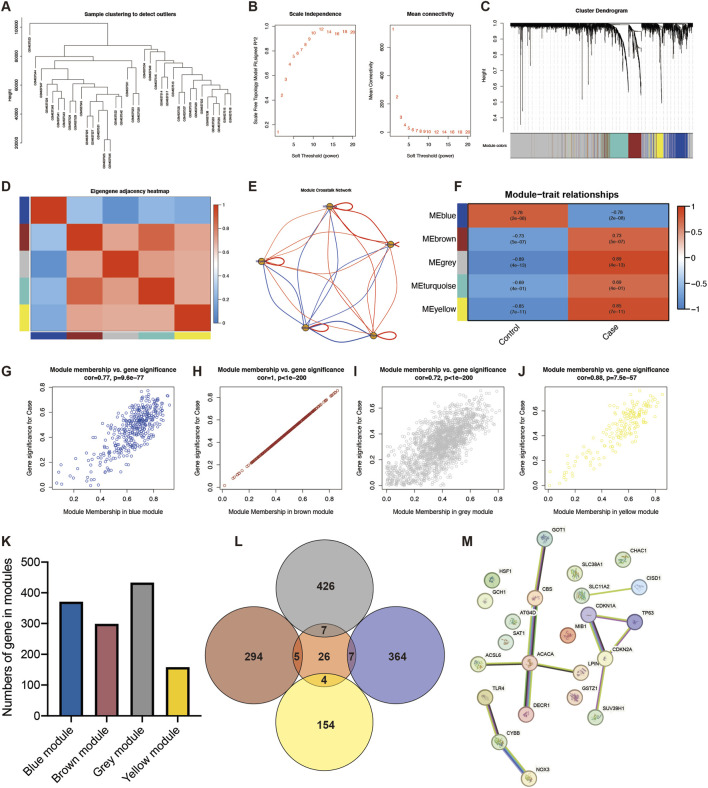
Gene co-expression construction. **(A)** Sample clusters. **(B)** Soft-thresholding. **(C)** Gene dendrogram and modules. **(D)** Module eigengenes with color labels. **(E)** Module crosstalk network. **(F)** Correlation analysis of modules with clinical trait. Correlation of gene significance (GS) and module membership (MM) in blue **(G)**, brown **(H)**, gray **(I)**, and yellow modules **(J)**. **(K)** Numbers of genes in modules. **(L)** Venn diagram illustrating overlapping of module genes and hub FRGs. **(M)** Protein–protein interaction network.

WGCNA was performed to identify gene modules associated with sarcopenia. Initial sample clustering (Figure 3A) revealed one sample as a significant outlier based on inter-sample Euclidean distance. This outlier was excluded to prevent it from distorting network topology, and the subsequent module construction was carried out using the remaining 35 samples (Figure 3A). Subsequent to the construction of a co-expression network, soft-thresholding was determined beta (β) = 12 (Figure 3B). Five modules were screened out (Figure 3C). The heatmaps illustrate the correlation of modules (Figure 3D). The crosstalk network of modules is demonstrated in Figure 3E. Among these were four relevant modules that were screened encompassing blue (r = −0.78, P = 2e-08), brown (r = 0.73, P = 5e-07), gray (r = 0.89, P = 4e-13), and yellow modules (r = 0.85, P = 7e-11) (Figure 3F). Gene significance (GS) and module membership (MM) also illustrated a significant relationship comprising blue (r = 0.77, P = 9.6e-77, Figure 3G), brown (r = 1.0, P < 0.001, Figure 3H), gray (r = 0.72, P < 0.001, Figure 3I), and yellow modules (r = 0.88, P = 7.5e-57, Figure 3J). The numbers of genes in modules are suggested in Figure 3K. A Venn diagram selected the intersection between modules and novel genes (Figure 3L). These intersection genes were visualized by protein–protein network (Figure 3M).

### Candidate key gene identification through machine learning algorithms and ROC curves

**FIGURE 4:**
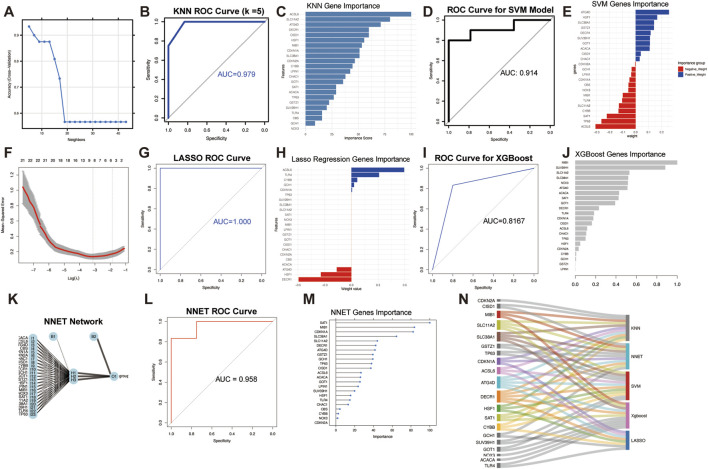
Screening key genes by multiple machine learning algorithms. **(A)** Best k value. **(B)** ROC curve of KNN model. **(C)** Gene significance of KNN model. **(D)** ROC curve of SVM model. **(E)** Gene significance of SVM model. **(F)** LASSO regression algorithm. **(G)** ROC curve of LASSO model. **(H)** Gene significance of LASSO model. **(I)** ROC curve of XGBoost model. **(J)** Gene significance of XGBoost model. **(K)** NNET network. **(L)** ROC curve of NNET model. **(M)** Gene significance of NNET model. **(N)** Sankey plot depicting network of genes and different machine learning algorithms.

To select genes with great value for diagnostic efficiency, five machine learning algorithms were utilized to screen hub genes, encompassing KNN, SVM, LASSO regression, XGBoost, and NNET. Grounded in the more desirable k value (k = 5, Figure 4A), the AUC value of the KNN ROC curve was found to evaluate a noticeable model efficiency (AUC = 0.979, Figure 4B). Then, we selected 11 genes with more favorable importance values for further analysis in accordance with the median importance value (median = 42.2, Figure 4C). The SVM algorithm constructed the models with remarkable performance, with AUC = 0.914 (Figure 4D). The genes’ importance is revealed in Figure 4E. There were 12 genes with more satisfactory importance values in the SVM model with a median importance value (median = 0.11). The AUC values for LASSO regression were 1.0 (Figures 4F,G). In line with the minimum lambda value (0.0003372709), eight genes were extracted in the LASSO model (Figure 4H). Next, XGBoost screened 12 genes based on median value (0.028), with an AUC value of 0.8167 (Figures 4I,J). The NNET network was constructed by utilizing the NNET model (Figure 4K) with a high AUC value (AUC = 0.958, Figure 4L). We obtained 11 genes as candidate biomarkers in accordance with the median value of 26.68 (Figure 4M). Finally, a total of ten hub genes (*SLC11A2*, *SLC38A1*, *MIB1*, *CDKN1A*, *ACSL6*, *ATG4D*, *DECR1*, *HSF1*, *SAT1*, and *CYBB*) were greater than three machine learning models, which were used for further analysis (Figure 4N).

The performance of five machine learning models was subsequently validated by employing the GSE9103 dataset (Supplementary Figures 1A–E). As the above findings illustrate, KNN (AUC = 0.833) and XGBoost (AUC = 0.829) achieved notably high AUC values, while SVM (AUC = 0.75), LASSO regression (AUC = 0.719), and NNET (AUC = 0.725) exhibited moderate AUC performance. In the selection of diagnostically relevant genes, the feasibility and effectiveness of applying machine learning approaches can be collectively validated by these findings.

**FIGURE 5:**
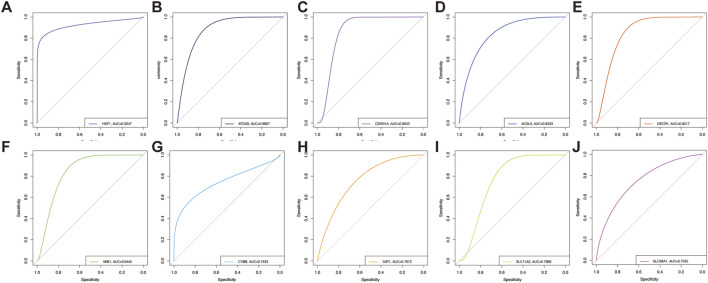
Hub genes analyzed by employing ROC curves. ROC curves of key FRGs, comprising *HSF1* **(A)**, *ATG4D* **(B)**, *CDKN1A* **(C)**, *ACSL6* **(D)**, *DECR1* **(E)**, *MIB1* **(F)**, *CYBB* **(G)**, *SAT1* **(H)**, *SLC11A2* **(I)**, and *SLC38A1* **(J)**.

In the evaluation of predictive performance, HSF1 demonstrated the highest AUC value (AUC = 0.9247, Figure 5A), which revealed its superior discriminatory power. Several genes also exhibited high AUC values of 0.8–0.9: *ATG4D* (AUC = 0.8807, Figure 5B), *CDKN1A* (AUC = 0.8643, Figure 5C), *ACSL6* (AUC = 0.8433, Figure 5D), *DECR1* (AUC = 0.8517, Figure 5E), and *MIB1* (AUC = 0.8445, Figure 5F). Genes featured by moderate AUC values (0.7–0.8) suggested a relatively weaker predictive capacity in this context. These distinctive genes encompassed *CYBB* (AUC = 0.7433, Figure 5G), *SAT1* (AUC = 0.7612, Figure 5H), *SLC11A2* (AUC = 0.7686, Figure 5I), and *SLC38A1* (AUC = 0.7525, Figure 5J).

### Potential drugs targeting diagnostic genes and molecular docking

**FIGURE 6:**
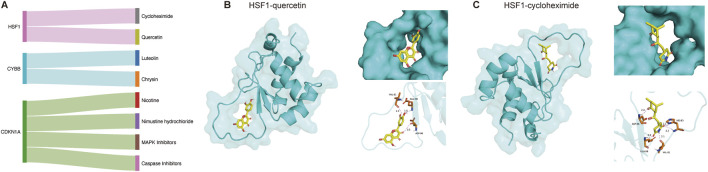
Gene–drug network in DGIdb database and molecular docking. **(A)** Sankey plot presenting drug–gene network. **(B)** Molecular docking between HSF1 and quercetin. **(C)** Molecular docking between HSF1 and cycloheximide.

Potential drugs targeting the biomarkers were identified by the DGIdb, which was predominantly intended to explore the potential drugs in sarcopenia. Two drugs targeting HSF1, two targeting CYBB, and drugs targeting CDKN1A were mined (Figure 6A). Subsequently, we performed molecular docking to assess the potential binding between HSF1 and the identified drug molecules, such as quercetin and cycloheximide. PyMOL software was utilized to visualize the results (Figures 6B,C). The binding energies of HSF1 to quercetin and cycloheximide were determined to be −6.0 and −5.8 kcal/mol, respectively.

### Effect of HSF1 on myoblast atrophy and ferroptosis-relevant markers

**FIGURE 7:**
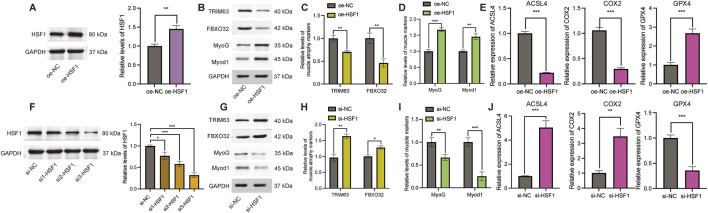
Effects of HSF1 on the expression of muscle atrophy- and ferroptosis-relevant markers. **(A)** Efficiency of HSF1 overexpression in C2C12 cells. **(B)** Representative Western blot images revealing protein levels of TRIM63, FBXO32, MyoG, and MyoD1 in the aftermath of HSF1 overexpression. **(C)** Quantitative analysis of TRIM63 and FBXO32 protein expression under HSF1 overexpression conditions. **(D)** Quantitative analysis of MyoG and MyoD1 protein expression subsequent to HSF1 overexpression. **(E)** Effects of HSF1 overexpression on ferroptosis-relevant markers (ACSL4, COX-2, and GPX4). **(F)** Efficiency of HSF1 knockdown via siRNA. **(G)** Representative Western blot images depicting protein levels of TRIM63, FBXO32, MyoG, and MyoD1 subsequent to HSF1 silencing. **(H)** Quantitative analysis of TRIM63 and FBXO32 expression in the aftermath of HSF1 knockdown. **(I)** Quantitative analysis of MyoG and MyoD1 expression in the wake of HSF1 silencing. **(J)** Effects of HSF1 knockdown on ferroptosis-relevant markers (ACSL4, COX-2, and GPX4). Quantification data for panels **(E, J)** derived from RT-PCR results and expressed as mean ± SD (n = 3). Data presented as mean ± SD; *P < 0.05, **P < 0.01, and ***P < 0.001.

In this section, overexpression vectors were performed to explore whether the HSF1 gene can affect C2C12 myoblast differentiation. The overexpression efficiency of the *HSF1* gene (oe-HSF1) was performed using Western blot analysis (Figure 7A) and RT-PCR measurements (Supplementary Figure 2A). The oe-HSF1 not only inhibited the expression of muscle atrophy markers TRIM63 and FBXO32 (Figures 7B,C) but also facilitated the expression of MyoG and Myod1 (Figures 7B,D). The overexpression of HSF1 remarkably downregulated the expression of key ferroptosis-relevant genes, encompassing ACSL4 and COX-2, while upregulating the expression of GPX4 (Figure 7E). After that, we used siRNA to perform targeted silencing of the HSF1 gene. As shown in Figure 7F, the interference efficiency of HSF1 was confirmed using Western blot analysis. To validate the knockdown efficiency of HSF1 at the transcriptional level, RT-PCR was performed on C2C12 cells transfected with three distinct HSF1-targeting siRNAs (si1-HSF1, si2-HSF1, and si3-HSF1). As shown in Supplementary Figure 2B, all three siRNAs significantly reduced HSF1 mRNA expression compared to the negative control siRNA (si-NC), with si3-HSF1 exhibiting the highest interference efficiency (P = 5.09615E-07). Based on this result, si3-HSF1 was selected for all subsequent functional experiments.

As is evident from the Western blot findings, the expression of muscle atrophy markers TRIM63 and FBXO32 in the silenced group was more satisfactory than that in the control group (Figures 7G,H), while silencing HSF1 decreased the expression of muscle marker protein MyoG and Myod1 (Figures 7G,I). Accompanied by a marked reduction in GPX4 expression (Figure 7J), the silencing of HSF1 resulted in elevated mRNA levels of ACSL4 and COX-2, suggesting that HSF1 acts as a critical regulator of ferroptosis in muscle cells.

### Effect of HSF1 on myoblast lipid metabolism

**FIGURE 8:**
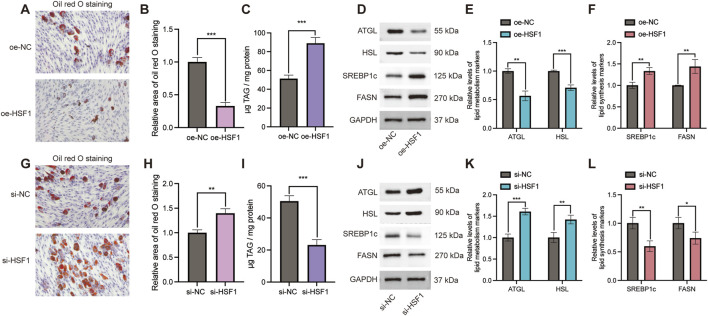
Effects of HSF1 on lipid metabolism in C2C12 cells. **(A)** Representative Oil Red O (ORO) staining of lipid droplets in cells overexpressing HSF1. **(B)** Quantitative analysis of ORO staining intensity under HSF1 overexpression conditions. **(C)** Intracellular triglyceride levels in the wake of HSF1 overexpression. **(D)** Representative Western blot images depicting expression of lipid metabolism-correlated proteins (ATGL and HSL) and lipid synthesis-associated proteins (SREBP1c and FASN) in aftermath of HSF1 overexpression. **(E)** Quantitative analysis of lipid metabolism-correlated protein expression in HSF1-overexpressing cells. **(F)** Quantitative analysis of lipid synthesis-associated protein expression in HSF1-overexpressing cells. **(G)** Representative ORO staining of lipid droplets in HSF1-silenced cells. **(H)** Quantitative analysis of ORO staining intensity subsequent to HSF1 knockdown. **(I)** Intracellular triglyceride levels following HSF1 silencing. **(J)** Representative Western blot images exhibiting expression of lipid metabolism- and synthesis-associated proteins in the wake of HSF1 knockdown. **(K)** Quantitative analysis of lipid metabolism-correlated protein expression in HSF1-silenced cells. **(L)** Quantitative analysis of lipid synthesis-associated protein expression in HSF1-silenced cells. Data presented as mean ± SD; *P < 0.05, **P < 0.01, and ***P < 0.001.

To further investigate the role of HSF1 in lipid metabolism, ORO was utilized to detect lipid content. As demonstrated by ORO staining with a low percentage of ORO-positive cells (Figures 8A,B) and triglyceride (TAG) quantification with more desirable TAG content (Figure 8C), the overexpression of HSF1 remarkably enhanced intracellular lipid accumulation. Furthermore, HSF1 upregulation is associated with a significant reduction in the expression of key lipid metabolism genes, including ATGL and HSL (Figures 8D,E) while promoting SREBP1c and FASN proteins involved in lipid synthesis (Figures 8D,F). Additionally, HSF1 knockdown illustrated a remarkable percentage of ORO-positive cells and big lipid droplets (Figures 8G,H). Importantly, a significant reduction was observed in intracellular triglyceride content (Figure 8I). In the wake of HSF1 knockdown, the expression of lipid metabolism-correlated proteins (ATGL and HSL) were strikingly elevated (Figures 8J,K), but the expression of lipid synthesis-associated genes (SREBP1c and FASN) was decreased in Western blot results (Figures 8J,L).

## Discussion

As a recently recognized musculoskeletal disorder, sarcopenia has emerged as a highly prevalent condition among aging populations, with severe consequences encompassing elevated frailty, mobility impairment, and heightened mortality risk (Cruz-Jentoft and Sayer, 2019; Gielen et al., 2023). While ferroptosis has been implicated in diverse biological processes through its regulation of iron-dependent lipid peroxidation, it remains poorly understood with regard to the diagnostic potential of ferroptosis-relevant genes (FRGs) and their mechanistic contributions to sarcopenia pathogenesis. Our comprehensive and systematic study explores novel ferroptosis-associated molecular biomarkers to address this critical knowledge gap. By carrying out comprehensive bioinformatics analysis and experimental validation, we identify key ferroptosis regulators that may serve as both diagnostic indicators and therapeutic targets. Moreover, we elucidate the potential molecular mechanisms by which these genes influence sarcopenia progression, particularly their roles in muscle atrophy pathways and lipid metabolism pathways. These findings not only advance our understanding of ferroptosis in age-correlated muscle deterioration but also lay a solid framework for developing targeted interventions against this debilitating condition.

This study identified a total of 23 FRGs by WGCNA and protein–protein network. Subsequently, we obtained ten key genes with remarkable gene significance in multiple machine learning algorithms. Using AUC>0.8 as the threshold, receiver operating characteristic (ROC) curves illustrated six genes (*HSF1*, *ATG4D*, *CDKN1A*, *DECR1*, *ACSL6*, and *MIB1*) with more satisfactory performance. HSF1 illustrated the highest AUC value, which evidently underscores the best diagnostic potential. In the aftermath of the above steps, we further identified quercetin and cycloheximide as promising agents for patients suffering from sarcopenia through the drug–gene interaction database (DGIdb) and molecular docking targeting HSF1. As a prognostic and diagnostic biomarker of gastric cancer (Kim and Kim, 2021), HSF1 served as a potential diagnostic biomarker in the treatment of endometriosis patients (Pan and Wan, 2023). Similarly, our results demonstrated more satisfactory diagnostic performance (AUC = 0.9247, Figure 5A). Consistent with our results, Jia et al. (2025) proposed that suppressing HSF1 expression resulted in inhibiting fatty acid synthesis. The depletion of HSF1 led to marked rearrangements of the organismal lipid landscape (Oleson et al., 2024). Of note, our findings stand in contrast to previously reported data in murine hepatic systems, where HSF1 overexpression was demonstrated to stimulate lipid metabolism and consequently lessen lipid droplet accumulation (Pan et al., 2021). In certain studies, HSF1 was identified as a central regulator of cellular bioenergetics and protein homeostasis (Qiao et al., 2017). The activation of HSF1 in smooth muscle cells has been found to upregulate the rate-limiting enzyme in cholesterol biosynthesis, thereby promoting cholesterol synthesis (Majumder et al., 2023). ATG4D has been acknowledged as a favorable diagnostic factor in Crohn’s disease (Brinar et al., 2012), cervical cancer (Meng et al., 2021), and colorectal cancer (Gil et al., 2018). Our results screened ATG4D as diagnostic gene in sarcopenia. ATG4 family proteins are cysteine proteases; more importantly, they can play pivotal roles in the lipidation and delipidation of LC3 during the autophagy process (Zhang et al., 2016). CDKN1A was obtained as a potential diagnostic biomarker in multiple diseases: acute myocardial infarction (Miao et al., 2023), keratoconus (Wang L. et al., 2022), and autism spectrum disorder (Cui et al., 2025). For now, no more than one published study has identified CDKN1A as a biomarker with diagnostic value for sarcopenia (Chen et al., 2024), demonstrating both desirable sensitivity and specificity. This finding was strongly corroborated by our results (Figure 5C). DECR1 was employed as an unfavorable biomarker and was bound up with immune infiltration in cervical cancer (Guo et al., 2023). DECR1 also had favorable sensitivity and specificity for distinguishing patients suffering moderate acute malnutrition in Sierra Leone (Singh et al., 2021). DECR1 is involved in the fatty acid beta-oxidation pathway as an auxiliary enzyme, which lowered steady-state levels of *de novo* fatty acid synthesis (Ursini-Siegel et al., 2007). The deletion of DECR1 prevented hypertrophy—mitochondrial dysfunction in cardiomyocytes—suggesting its potentiated as more satisfactory in mitochondrial lipid oxidation and cardiac damage (Lu et al., 2025). ACSL6 had high diagnostic value in non-small-cell lung cancer (Ma et al., 2024) and colon adenocarcinoma (Parsazad et al., 2023). ACSL6 is a key enzyme regulating the partitioning of acyl-CoA species toward different metabolic fates (Teodoro et al., 2017). ACSL6 was utilized to construct lipid metabolism-correlated signature in hepatocellular carcinoma (Hu et al., 2020). In rat myotubes, ACSL6 knockdown decreased the accumulation of TAGs and lipid droplets, suggesting that ACSL6 drove acyl-CoA toward lipid synthesis (Teodoro et al., 2017). ACSL6 contributed to lipid synthesis by converting docosahexaenoic acid (DHA) into DHA-CoA—a substrate during DHA-containing lipid biosynthesis (Kuroha et al., 2023). Notwithstanding the fact that current studies have validated the positive correlation between muscle ACSL6 protein abundance and intramuscular triglyceride content (Stierwalt et al., 2021), no comprehensive studies have been reported into how ACSL6 associates with lipid metabolism in the context of sarcopenia; our study identified ACSL6 as a novel diagnostic biomarker for sarcopenia. Consequently, our team will further investigate the functional role and underlying mechanisms of ACSL6 in sarcopenia pathogenesis. As of yet, no reports have been released to probe deeply into MIB1 in the context of sarcopenia. It is noteworthy that MIB1 has been established as a diagnostic biomarker in other diseases (Schmitt et al., 2006; Tarighati et al., 2023), which aligns with our own results. By carrying out immunohistochemistry, MIB1 was advantageous for the differentiation of benign from malignant adrenocortical tumors (Schmitt et al., 2006).

Emerging evidence suggests that as an iron-dependent form of regulated cell death characterized by excessive lipid peroxidation, ferroptosis contributes substantially to the progression of sarcopenia (Ru et al., 2025). The molecular mechanisms underlying this involvement are closely linked to dysregulated lipid metabolism (Zhang Y. et al., 2022). In our study, we systematically screened ferroptosis-relevant genes and identified six that demonstrated significant diagnostic value for sarcopenia. As revealed by our functional experiments, silencing HSF1 not only enhanced lipid metabolism but also facilitated the expression of established ferroptosis markers. This phenomenon demonstrated a regulatory role for HSF1 in ferroptotic cell death within muscle cells. This finding aligns with previous literature; for instance, TXNIP has been reported to promote ferroptosis in muscle satellite cells by disrupting glutathione metabolic pathways. Under such circumstances, it can ultimately contribute to muscle degeneration (Maimaiti et al., 2025). Driven by the accumulation of phospholipid hydroperoxides, ferroptosis was believed to result from an imbalance between polyunsaturated fatty acid (PUFA)-containing phospholipids synthesis and the antioxidant capacity of the glutathione (GSH)–glutathione peroxidase 4 (GPX4) axis (Sokol et al., 2025). This was tremendously evident in cellular membranes. In skeletal muscle, this process may result in the loss of mitochondrial integrity, atrophy, and impaired muscle function (Xiang et al., 2025). Altered lipid metabolism in sarcopenia, encompassing elevated lipid storage and peroxidation, also creates a microenvironment advantageous for ferroptosis (Chen et al., 2024). The depletion of GPX4 and subsequent iron overload amplify oxidative damage, thereby accelerating muscle wasting and functional decline in age-correlated sarcopenia (Wang Y. et al., 2022).

Our findings on the role of HSF1 in regulating lipid metabolism and ferroptosis in sarcopenia are further supported and extended by Zhang et al. (2025), who demonstrate that skeletal muscle-specific HSF1 activation alleviates age-associated sarcopenia and mitochondrial decline through the SIRT3–PGC1α axis. While they focused on mitochondrial biogenesis and function and we highlight lipid dysregulation and ferroptosis, we both converge on HSF1 as a critical protective regulator in aging muscle. The activation of the SIRT3–PGC1α pathway by HSF1, as shown by Zhang et al. (2025), could potentially intersect with our observed metabolic outcomes, as PGC1α is a well-known master regulator of cellular metabolism. Together, these studies underscore the multifaceted therapeutic potential of targeting HSF1 to combat sarcopenia through distinct yet potentially synergistic mechanisms, improving mitochondrial health and preventing lipid peroxidation-driven ferroptosis.

While we have identified HSF1 as a high-performance diagnostic biomarker for sarcopenia based on computational and *in vitro* evidence, its clinical applicability and translational potential warrant further consideration. The high AUC value (>0.92) suggests strong discriminatory power in our analyzed datasets, primarily derived from blood or tissue transcriptomics. For potential translation into a clinical diagnostic tool, future studies are needed to validate HSF1 expression levels in easily accessible human biofluids (e.g., serum or plasma) from well-characterized cohorts of sarcopenia patients and controls using standardized assays such as ELISA or quantitative PCR. Furthermore, correlating HSF1 levels with clinically relevant parameters (e.g., muscle mass, strength, and physical performance) would be essential for establishing its utility as a practical biomarker. Beyond diagnosis, given its central regulatory role in lipid metabolism and ferroptosis pathways elucidated here, HSF1 also represents a promising candidate for therapeutic targeting. Modulating HSF1 activity or its downstream effectors could offer a novel strategy for counteracting muscle degeneration, moving from diagnostic biomarker potential to therapeutic intervention.

Notwithstanding potential breakthroughs contributed by our study, we acknowledge several limitations. Above all, the limited clinical information of public data potentially triggered biased analytical results attributable to the risk of sample contamination. In addition, as a consequence of limitations in our project funding, the efficacy and safety of the identified potential drugs lacked further validation.

While this study identifies HSF1 as a high-performance diagnostic biomarker for sarcopenia based on integrated bioinformatics and *in vitro* experimental evidence, its translation into a clinical setting requires a defined pathway to address the inherent limitations of our current work. First, to move beyond the potential biases associated with public data, prospective validation in well-characterized, independent human cohorts is essential. This involves measuring HSF1 expression (mRNA or protein) in easily accessible biofluids (such as serum or plasma) from sarcopenia patients and age-matched controls using standardized clinical assays (ELISA). Crucially, these HSF1 levels must be rigorously correlated with gold-standard clinical metrics of sarcopenia (e.g., muscle mass via DEXA, grip strength, and gait speed). Second, regarding therapeutic potential, the promising drugs identified through our molecular docking analysis require comprehensive *in vivo* validation of their efficacy and safety in relevant preclinical models of sarcopenia—necessary before any clinical consideration. Thus, HSF1 emerges from this study not as a ready-to-use clinical tool but as a compelling and mechanistically grounded candidate. The logical next steps—targeted clinical validation and rigorous *in vivo* drug testing—are clearly defined and would directly address the current limitations, paving the way for its potential future application in diagnosis and targeted therapy for sarcopenia.

## Conclusion

By using integrative bioinformatics and experimental approaches, this study systematically identified and validated a set of ferroptosis-relevant genes (*HSF1*, *ACSL6*, *CDKN1A*, *ATG4D*, *DECR1*, and *MIB1*) as diagnostic biomarkers for sarcopenia. We ultimately concluded that HSF1 not only serves as a promising diagnostic indicator but also functionally regulates lipid metabolism and ferroptosis in muscle cells. These findings reinforce our understanding of the molecular mechanisms linking ferroptosis to sarcopenia. They also lay a robust foundation for future research into targeted therapies. Despite limitations in clinical sample availability and drug validation, our study offers novel insights and candidate biomarkers that may facilitate early diagnosis and precision treatment of sarcopenia. Further investigation into the roles of these FRGs *in vivo* and in clinical cohorts is warranted to translate these findings into therapeutic applications.

## Data Availability

The datasets presented in this study can be found in online repositories. The names of the repository/repositories and accession number(s) can be found in the article/Supplementary Material.

## Supplementary material

SUPPLEMENTARY FIGURE 1 Validation of machine learning algorithms in the GSE9103 dataset. ROC curve in KNN model **(A)**, SVM model **(B)**, LASSO regression **(C)**, XGBoost model **(D)**, and NNET model **(E)**.

SUPPLEMENTARY FIGURE 2 Efficiency of HSF1 overexpression and knockdown derived from RT-PCR results. **(A)** HSF1 overexpression promoted mRNA expression of HSF1. **(B)** HSF1 knockdown inhibited mRNA expression of HSF1.
