# Supplementary figures and images for: HSF1 modulates lipid metabolism and ferroptosis in sarcopenia: a novel diagnostic biomarker and therapeutic target
Source: Front Mol Biosci. 2026 Apr 30;13:1727633. doi: 10.3389/fmolb.2026.1727633 (PMC13172621; doi:10.3389/fmolb.2026.1727633)

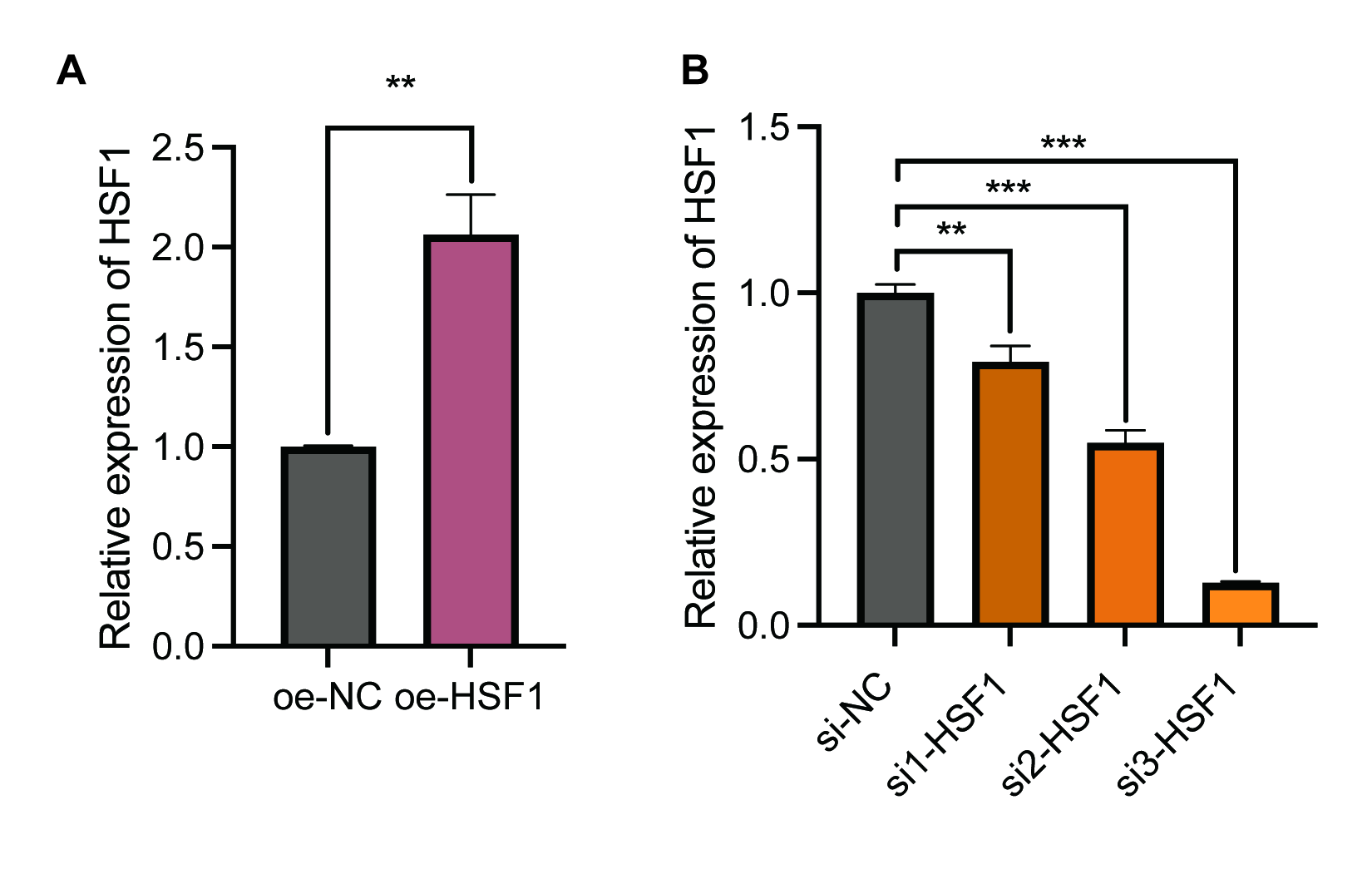

Supplement: Supplementary file 1 [file Image2.tif]

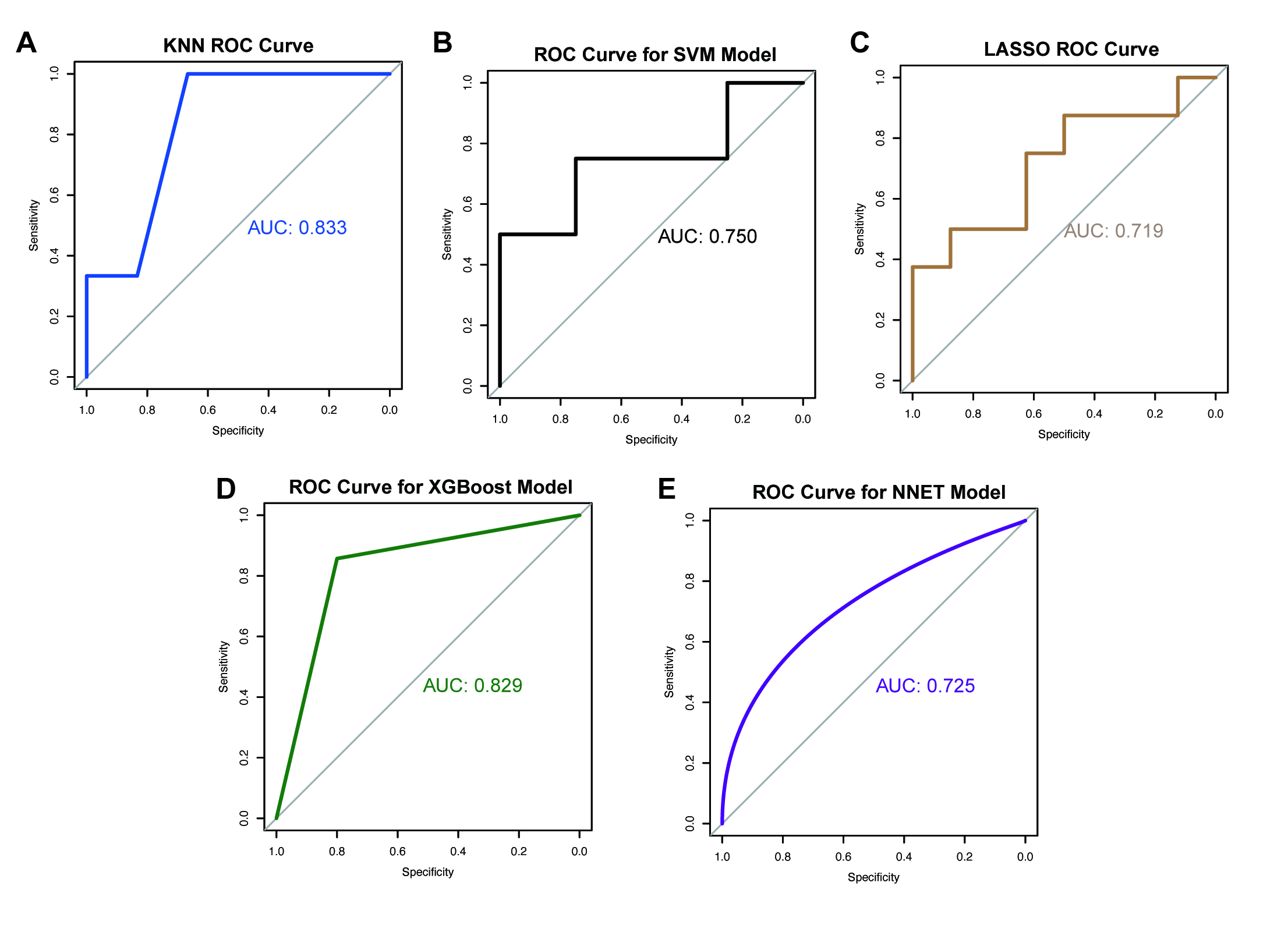

Supplement: Supplementary file 2 [file Image1.tif]
