# Supplementary material for: HSF1 modulates lipid metabolism and ferroptosis in sarcopenia: a novel diagnostic biomarker and therapeutic target
Source: Front Mol Biosci. 2026 Apr 30;13:1727633. doi: 10.3389/fmolb.2026.1727633 (PMC13172621; doi:10.3389/fmolb.2026.1727633)
Supplement: Supplementary file 3 [file Table2.docx]

Supplementary table 2. The primer sequences for quantitative real-time PCR.

| Target genes | Sequence |
| --- | --- |
| GPX4 | F: 5’-GCCTCGCAATGAGGCAAAAC-3’ |
| GPX4 | R: 5’-CAAACTGGTTGCAGGGGAAG-3’ |
| COX2 | F: 5’-CAGGACTCTGCTCACGAAGG-3’ |
| COX2 | R: 5’-ATCCAGTCCGGGTACAGTCA-3’ |
| ACSL4 | F: 5’-CCGACCTAAGGGAGTGATGAT-3’ |
| ACSL4 | R: 5’-CAGAGAGTGTAAGCGGAGAAGAA-3’ |
| HSF1 | F: 5’-TACCCAAGTACTTCAAGCACA-3’ |
| HSF1 | R: 5’-CAGTGACCACTTGGATGCTAT-3’ |
| GAPDH | F: 5’-TGCACCACCAACTGCTTAG-3’ |
| GAPDH | R: 5’-GGATGCAGGGATGATGTTC-3’ |
